# Roads and bats: a meta‐analysis and review of the evidence on vehicle collisions and barrier effects

**DOI:** 10.1111/mam.12072

**Published:** 2016-05-29

**Authors:** Amy Grace Fensome, Fiona Mathews

**Affiliations:** ^1^ Hatherly Laboratories, BiosciencesCollege of Life and Environmental SciencesUniversity of ExeterPrince of Wales RoadExeterEX4 4PSUK

**Keywords:** barrier effect, bats, habitat fragmentation, meta‐analysis, roads

## Abstract

1. Roads are a potential threat to bat conservation. In addition to the direct risk of collision of bats with vehicles, roads could pose a threat to bat populations as a result of habitat loss, degradation and fragmentation, and could act as barriers to movements of bats between habitats.
2. We performed a systematic review of the literature and conducted meta‐analyses to assess the threat posed by roads to bats as a result of 1) collisions between bats and vehicles and 2) roads acting as barriers to movements of bats.
3. Based on collated records of 1207 bat road casualties in Europe, we found that low‐flying species are more prone to collisions than high‐flying species, and that juveniles are more vulnerable to collisions than adults. In addition, meta‐analysis identified a significant bias towards male casualties. Casualties included rare species such as *Barbastella barbastellus* and geographically restricted species such as *Rhinolophus* species.
4. The bias towards male casualties could be indicative of greater natal philopatry or lower dispersal among females, or of sexual segregation in habitats of varying quality, i.e. females may occupy better quality habitats than males, and road density may be lower in better quality habitats.
5. Whether or not roads act as barriers to the movement of bats depends on a complex interplay of habitat and species‐specific behaviour. For example, the presence of favourable habitat for bats – notably woodland – was found in this review to be linked with significantly reduced barrier effects but a heightened risk of collision.
6. Our data suggest that roads do pose a threat to bats. Future research should assess the contribution of traffic noise and street lighting to the barrier effect of roads. Where new road schemes are monitored by ecological practitioners, it is vital that consistent protocols are employed to ensure that bat activity can be compared before and after the road is built. Evidence from such research should be used to minimize the risks for bats of any roads built in the future, and to design safe crossing points for bats.

## Introduction

The road network is expanding rapidly throughout Europe. Between 2004 and 2013, over 630000 km of new roads were built, an average of at least 70000 km each year (Anonymous 2013). The network will expand further during the next 5 years as the European Commission oversees the investment of €24 billion in the transport network by 2020 (Anonymous 2014a), while the UK government plans to invest more than £15 billion on 127 road‐building projects, including 400 additional miles of motorway and major road widening schemes (Anonymous 2014b).

There are several life‐history traits which place bats in general at high risk from roads. Requiring a larger home range than would be predicted for a mammal of their body mass (Kelt & Van Vuren 1999), they may be particularly sensitive to the loss and fragmentation of habitat. Their use of different locations for foraging, mating, hibernating, and breeding, and their movements between summer roosts and hibernation roosts (Schofield & Mitchell‐Jones 2011) are also likely to elevate bat encounter rates with roads. Bats are very long lived, and have low fecundity (1–2 offspring per year; Kunz & Fenton 2003), so may be unable to withstand even moderate increases in mortality (Schorcht et al. 2009). In addition, many live at low densities and have a patchy distribution, making them vulnerable to local extinction.

We examine the available evidence that roads pose a threat to bat populations as a result of collisions between bats and vehicles, and by obstructing movement across roads. How bats interact with roads (i.e. the extent to which they exhibit avoidance behaviour) determines whether and how a road poses a threat. These interactions are likely to be influenced by a number of factors including the behaviour and ecology of bats, characteristics of the road, such as width and traffic volume and the habitat characteristics in the vicinity of the road.

Within species, there may also be differences in collision risk. We predict elevated risks in juveniles due to their inexperience of orientation, and in encountering vehicles and artificial lights. Juveniles may also have slower and less manoeuvrable flight, as bats with high manoeuvrability tend to have low wing loadings, and it has been shown in *Myotis lucifugus* that wing loading declines as juvenile bats grow (Adams 1996). It is also possible that juvenile bats prefer to forage or practice flying in the open areas provided by roads. Habitat use has been shown to vary with age. For example, juvenile *Myotis lucifugus* are more likely to forage in less cluttered habitats than adults (Adams 1997).

There may also be sex differences in collision susceptibility, and the expected effects may vary according to the time of year. Females could have a higher risk of collision during late spring and early summer, when pregnancy and lactation means they are heavier and less manoeuvrable, need to forage earlier and for longer, and make regular returns to the roost to feed young. Conversely, in swarming species, males are likely to be more susceptible to collision than females in the autumn, because of the strong bias towards males visiting swarming sites, especially earlier in the season (Parsons et al. 2003). Assuming a 1:1 sex ratio within a population as a whole, male bats must visit more swarming sites than females and must make more journeys in order to access these sites.

Many bat species use linear features such as hedgerows and treelines to orient themselves between roosts and foraging sites (Altringham 2011) or for foraging (Downs & Racey 2006). We predict that the presence of hedges or trees running parallel with or perpendicular to a road could enhance the road's permeability to bats, but also increase the likelihood of collisions between bats and vehicles, by encouraging bat commuting or foraging activity.

Road width is likely to be negatively correlated with permeability for a number of species: low‐flying bats that tend to forage or commute within closed environments (e.g. woodland) may be less likely to cross wide roads than species adapted to flying high or to using open landscapes. Roadside lighting is similarly expected to have a species‐specific effect. Light is thought to deter slow‐flying species (Stone et al. 2009) whereas other species, such as *Pipistrellus pipistrellus,* use lit roads provided that tree cover is available (Blake et al. 1994, Mathews et al. 2015). The risk of collision for such species depends on the height of the lights and the height at which individuals commute to and from the site.

The extent to which traffic might repel bats from the vicinity of roads is less clear, but is likely to vary between species, according to their responsiveness to light and noise, and may depend on traffic volume. Bats are thought to avoid locations with loud background noise, as it interferes with their ability to use acoustic information to locate insects, thereby reducing foraging efficiency (Schaub et al. 2008, Siemers & Schaub 2011), and it may also affect commuting behaviour. Moving traffic may also be perceived as a threat and induce predator avoidance behaviours (Baxter et al. 2006, Zurcher et al. 2010). These factors are not mutually exclusive and could deter bats from the vicinity of roads. Alternatively, high traffic volumes could simply increase the risk of collisions between bats and vehicles.

We systematically review the literature and, where data allow, conduct meta‐analyses to assess the threat posed to bats by roads through collisions and as barriers to movement. We examine the composition of collated records of road‐killed bats to determine whether flight height, sex or age of individuals influences collision risk, and we present data on the temporal and spatial distribution of mortalities. We assess the evidence that road permeability depends on a species' foraging ecology, and is influenced by characteristics of the road, such as width and traffic volume, as well as by the ecology and topology in the vicinity of the road.

## Evidence Base

A systematic search was made of the Web of Knowledge, EBSCO and Google Scholar in February and March 2015 using the search terms ‘(road*OR highway* OR motorway* OR vehicle*)’ and ‘(bat OR bats OR Chiroptera)’ with either ‘(fatal* OR mortal* OR collision*OR casualty*)’ or ‘(barrier OR permeability OR cross OR crossing)’. Through these searches, we identified 12 articles relating to bats as casualties on roads, and eight relating to the barrier effect of roads (Table 1). Due to the paucity of data from elsewhere, the review was restricted to Europe with the exception of two studies which were conducted in the USA (Russell et al. 2009, Kitzes & Merenlender 2014). Russell et al. (2009) provide data which were included in the meta‐analyses of sex and age. Sensitivity analyses confirmed that exclusion of this study from analyses had a minimal effect.

**Table 1:** Articles retrieved during our literature search relating to 1) collisions between bats and vehicles and 2) roads as barriers to the movements of bats. For road casualty data, where the information has been provided by the authors, we note the frequency and location of searches, whether these were conducted on foot, by car or by bike, and the dates between which surveys took place. We include the total number of carcasses reported by each author as well as the proportions of both sex and age classes of individuals where these data were available

Country	Method	Results	References
Collisions			
France	Roadside hedgerows searched weekly, on foot, May‐October 1998–2002 (24 months)	Total 109 bats found dead	Capo et al. (2006)
Czech Republic	Emergency stopping lanes searched approx. weekly, on foot, May‐October 2007 (6 months)	Total 119 bats found dead	Gaisler et al. (2009)
Spain	Lanes, hard shoulders and ditches searched weekly on foot, 1989 (12 months)	Total 72 bats found dead	Gonzalez‐Prieto et al. (1993)
Germany	Collated incidental records, 1945–1995	Total 307 bats found dead, 211 males and 96 females	Haensel and Rackow (1996)
Montenegro	Two roads searched weekly by bike, August‐October 2013 (3 months)	Total 17 bats found dead, 8 males and 5 females	Iković et al. (2014)
Germany	Collated incidental records, 1964–1993	Total 96 bats found dead	Kiefer et al. (1995)
Poland	Approx. weekly searches, May‐October 1994–2000 (36 months)	Total 167 bats found dead, 30 males and 31 females, 56 juveniles and 29 adults	Lesiński (2007) Method 1
Poland	Irregular searches on several roads, 1992–1993 and 2001–2004		Lesiński (2007) Method 2
Poland	Roadsides, searched approx. weekly, August‐September 2004 and April‐October 2005–2006 (16 months)	Total 44 bats found dead, 9 males and 15 females	Lesiński (2008)
Poland	Roadside, verges and ditches searched weekly, by car and by foot, July 2008‐June 2009 (11 months)	Total 61 bats found dead, 20 males and 7 females, 17 adults and 8 juveniles	Lesiński et al. (2010)
Portugal	Daily search of several roads by car, March‐October 2009 (7.5 months)	Total 154 bats found dead, 44 males and 20 females, 99 adults and 17 juveniles	Medinas et al. (2012)
Germany	Collated incidental records, 1951–1993	Total 61 bats found dead	Rackow et al. (1994)
Pennsylvania, USA	Road and verges, searched approx. weekly, on foot May‐September 2001 (4 months)	Total 29 bats found dead, 4 males and 16 females, 12 adults and 15 juveniles	Russell et al. (2009)
Roads as barriers			
Ireland	Acoustic monitoring of bat activity at four types of motorway crossing: over‐road bridges (×6), severed treelines (×6), underpasses (×7) and river bridges (×6). Activity at crossing sites compared to that in adjacent landscape. Road width ~65–70 m, ~20000 vehicles per day	Under‐road routes preferred to over‐road routes. An average of 23.5 fewer bat passes at over bridges, 7 fewer at severed treelines, 19.5 more passes at underpasses, 158 more passes beneath river bridges than compared to adjacent sites	Abbott et al. (2012a,b)
Ireland	Acoustic monitoring of bat activity at three under‐road passageways of different dimensions: two narrow (*H* = 1.23 m, 1.1 m; *W* = 1.48 m, 1.4 m), one wide (*H* = 6 m, *W* = 16.6 m). Road width ~60–65 m, ~11000 vehicles per day	Clutter‐adapted species less likely to use over‐road routes than open or edge‐adapted species. Clutter‐adapted species were also more likely to use the narrow under‐road passages	Abbott et al. (2012a,b)
Indiana, USA	Crossing and avoidance behaviour of bats at five survey sites observed. Species identity, flight height, presence/absence of vehicles, and local ecology recorded	Bats were more likely to avoid crossing a road in the presence of vehicles, in the absence of trees, with lower flight height (See Appendices S9 and S10)	Bennett and Zurcher (2013)
England	Bat activity monitored acoustically at varying distances (0–1600 m) from a motorway, road width 35 m, 30–40000 vehicles per day	Species diversity and bat activity declined with proximity to the road. Bat activity at 1600 m was 3.5 times that at the road	Berthinussen and Altringham (2012b)
England	Road‐crossing behaviour monitored acoustically and by observers at four roads to compare frequency with which bats used underpasses, bat gantries and commuting routes. Flight height and verge height flew were also recorded	Bats more likely to cross roads at unsafe heights than to use underpasses. Few bats crossed at gantries but where they did, most flew at unsafe heights (≤5 m). The height at which bats flew over the road was strongly correlated with verge height	Berthinussen and Altringham (2012a)
Germany	Six *Barbastella barbastellus* (low flying and open adapted) and 34 *Myotis bechsteinii* (low flying and clutter adapted) were radio‐tracked. Mist‐netting conducted in three underpasses. Road width 18–23 m, 84000 vehicles per day	More *Barbastella barbastellus* (5/6) crossed the road than *Myotis bechsteinii* (3/34). Most *Barbastella barbastellus* crossed above the road (21/37); all *Myotis bechsteinii* crossed at underpasses (36/36). *Myotis bechsteinii* foraging ranges were smaller closer to the road and females with smaller foraging areas had lower reproductive success	Kerth and Melber (2009)
California, USA	Bat activity monitored acoustically at three sites, at incremental distances from the road. Road widths and traffic densities: 25–45 m and 55000 vehicles per day; 40 m and 86000 vehicles per day; 15 m and 33500 vehicles per day	Activity was approximately twice as high 300 m from a road as at the road	Kitzes and Merenlender (2014)
Indiana, USA	Road‐crossing behaviour (cross/avoid, flight height) at roads was observed at five sites and the presence/absence of vehicles, noise level emitted by vehicles and their speed were recorded	Vehicles present: 40% (28/44) of bats crossed the road. Vehicles absent: 58% (103/167) of bats crossed. Noise level, speed of the vehicle and flight height had no effect on the tendency for bats to cross	Zurcher et al. (2010)

From the studies relating to collisions, we extracted data on the species, sex and age of road‐killed bats, as well as the approximate date on which they were found. We also noted methodological information such as the length of the road surveyed and the duration of the study. The number of studies that took place within each species' range was recorded.

To facilitate comparisons between studies, casualty numbers are expressed as rates per kilometre of road per month. We were not able to calculate these figures for Germany, since data were derived from incidental records rather than systematic surveys of known stretches of road (Rackow et al. 1994, Kiefer et al. 1995, Haensel & Rackow 1996).

Where data were sufficient, meta‐analyses were conducted. We were able to conduct three separate analyses to examine potential differences in collision risk based on sex, age, and flight height. Analysis of variation in collision risk over time was not possible since the date of carcass collection was rarely reported.

To permit assessment of the links between flight height and road impacts, we assigned species to either high‐flying or low‐flying categories (the category to which each species was assigned is shown in Appendix S1). High‐flying species were defined as those usually flying more than 10 m above the ground (above the height of cars); low‐flying species were defined as those that typically fly up to 5 m from the ground (Russ 1999). Forty‐five individuals were not assigned to either category as they were not identified to species. Species in the genus *Pipistrellus* have variable flight heights but typically fly below 10 m (Russ 1999). They are also the most abundant and widespread group. For these reasons, we decided to conduct the analysis both with and without individuals from this genus; where they were included, we placed them in the low‐flying category.

Assuming that all adults in a population reproduce, and that most European bats produce singleton offspring, the expected ratio of adults to juveniles is 2:1, thus the expected proportion of juveniles within the population is 0.33 (this is a conservative estimate; in many populations the proportion will be lower than 0.33 due to some adults not breeding). The sex ratio was assumed to be 1:1 in all bat populations. The expected ratio of low‐flying to high‐flying species could not be determined due to lack of data, so it was not possible to test whether the observed ratio differed significantly from that predicted by their abundance in the environment.

Meta‐analyses were conducted in R version 3.1.2 (Anonymous 2014c) using the package ‘meta’ (Schwarzer 2015). The analyses were based on binomial data and therefore we used logit transformations and confidence intervals based on Wilson Scores. The sensitivity of the analyses to the exclusion of individual studies was tested.

In cases where insufficient data were available, or where there was large variability in the definition of exposures (e.g. in assessments of habitats and crossing points associated with casualty risks or barrier effects), a qualitative report of the literature is provided instead of meta‐analysis.

## Results

We collated 1207 records of bat road casualties spanning five decades. The country with the highest recorded number of casualties was Germany, where 464 individual bats were collated from records made over a 50‐year period. The lowest number, among countries from which data were available, came from Montenegro, where 17 bats were recovered from roads during 14 months. Monthly casualties ranged from 0.03 bats km^−1^ in Montenegro (Iković et al. 2014) to 2.5 bats km^−1^ in the Czech Republic (Gaisler et al. 2009, Appendix S2). Most studies took place within the ranges of the species most frequently recorded as casualties (Table 2). Methodological variations account for some of the observed differences between studies, but site characteristics were also influential (see Table 1 and Appendix S3).

**Table 2:** The extent to which the combined studies are representative of each species of bat found in Europe. As such, studies conducted in the USA have been excluded; •, study reported this species, ○, study took place within the geographical range of this species but no individuals were found; X, study took place outside this species' range

Species	Capo et al. (2006)	Gaisler et al. (2009)	Gonzalez‐Prieto et al. (1993)	Haensel and Rackow (1996)	Iković et al. (2014)	Kiefer et al. (1995)	Lesiński (2007)	Lesiński (2008)	Lesiński et al. (2010)	Medinas et al. (2012)	Rackow et al. (1994)	Number of studies within species' range	Number of studies where sp. found within range
	France	Czech Republic	Spain	Germany	Montenegro	Germany	Poland	Poland	Poland	Portugal	Germany		
*Rhinolophus blasii*	X	X	X	X	•	X	X	X	X	X	X	1/11	1/1
*Rhinolophus ferrumequinum*	*•*	○	○	•	○	○	○	○	○	•	○	11/11	3/11
*Rhinolophus hipposideros*	○	○	•	•	•	•	•	○	○	•	•	11/11	7/11
*Eptesicus nilsonii*	○	○	X	•	X	•	○	○	○	X	○	8/11	2/8
*Eptesicus serotinus*	○	•	○	•	○	•	•	○	•	•	•	11/11	7/11
*Nyctalus leisleri*	○	•	○	•	○	•	•	○	•	•	•	11/11	7/11
*Nyctalus noctula*	○	•	○	•	○	•	•	○	*•*	○	•	11/11	6/11
*Pipistrellus kuhlii*	*•*	X	○	○	*•*	○	○	○	○	*•*	○	10/11	3/10
*Pipistrellus nathusii*	○	*•*	○	*•*	*•*	○	*•*	○	*•*	○	○	11/11	5/11
*Pipistrellus pipistrellus*	*•*	*•*	*•*	*•*	○	○	○	○	○	*•*	*•*	11/11	6/11
*Pipistrellus pygmaeus*	○	*•*	○	○	*•*	○	○	○	○	*•*	○	11/11	2/11
*Pipistrellus savii*	○	X	*•*	○	○	○	X	X	X	○	○	7/11	1/7
*Myotis alcathoe*	○	•	○	○	X	○	○	○	○	X	○	9/11	1/9
*Myotis bechsteinii*	*•*	○	○	•	○	•	○	○	○	○	○	11/11	3/11
*Myotis brandtii*	○	•	X	•	○	•	•	○	○	X	•	10/11	5/9
*Myotis capaccinii*	○	X	○	X	•	X	X	X	X	X	X	3/11	1/3
*Myotis dasycneme*	○	○	X	○	X	○	•	○	○	X	○	8/11	1/8
*Myotis daubentonii*	*•*	*•*	*•*	○	○	•	•	○	○	•	•	11/11	7/11
*Myotis emarginatus*	*•*	•	○	○	○	○	○	○	○	○	○	11/11	2/11
*Myotis escalerai*	○	X	○	X	X	X	X	X	X	•	X	3/11	1/3
*Myotis nattereri*	*•*	•	○	•	○	•	•	•	•	○	•	11/11	8/11
*Myotis myotis*	*•*	○	○	•	○	•	•	○	○	○	•	11/11	5/11
*Myotis mysticanus*	*•*	○	○	•	•	•	•	○	○	○	○	11/11	5/11
*Barbastella barbastellus*	*•*	○	○	•	○	•	•	○	•	•	○	11/11	6/11
*Plecotus auritus*	*•*	○	○	•	○	○	•	•	•	○	•	11/11	6/11
*Plecotus austriacus*	*•*	○	•	•	○	○	•	○	○	○	•	11/11	5/11
*Vespertilio murinus*	○	○	X	•	○	○	○	○	○	X	•	9/11	2/9
*Miniopterus schreibersii*	○	X	○	•	○	•	○	○	○	•	○	10/11	3/10

It is evident that casualties are not evenly distributed temporally or spatially. Researchers consistently reported mortality peaks during the mating and swarming season of each species (Rackow et al. 1994, Kiefer et al. 1995, Haensel & Rackow 1996, Lesiński 2007, Gaisler et al. 2009, Lesiński et al. 2010, Medinas et al. 2012) or during migration to winter roosts (Medinas et al. 2012; Appendix S4).

## Collisions

### Risk factors associated with the behaviour and ecology of bats

Of the 1207 recorded casualties, most were from the genera *Pipistrellus* (35%, *n* = 419, from nine species) and *Myotis* (29%, *n* = 351, from 15 species; Table 3). Rarer species such as *Barbastella barbastellus* (2.3%, *n* = 28), which is undergoing declines in parts of its range, were also recorded (Anonymous 2015).

**Table 3:** The number of bat carcasses by species reported in each study. In total, 1207 bats were recovered from roads in Europe. The one study from the USA (Russell et al. 2009) that reported casualties was excluded. The number of months during which surveys took place is shown in parentheses after the country, except for the following papers which collated incidental records from multiple sources over the following periods: 1945–1995 (Haensel & Rackow 1996); 1964–1993 (Kiefer et al. 1995); 1951–1993 (Rackow et al. 1994)

Species	Capo et al. (2006)	Gaisler et al. (2009)	Gonzalez‐Prieto et al. (1993)	Haensel and Rackow (1996)	Iković et al. (2014)	Kiefer et al. (1995)	(2007)	(2008)	et al. (2010)	Medinas et al. (2012)	Rackow et al. (1994)	Total
	France (24)	Czech Republic (5)	Spain (12)	Germany	Montenegro (14.5)	Germany	Poland (36)	Poland (16)	Poland (11)	Portugal (7.5)	Germany	
*Rhinolophus blasii*					1							1
*Rhinolophus ferrumequinum*	2			1						1		4
*Rhinolophus hipposideros*			14	3	4	2	2			7	1	33
*Eptesicus nilsonii*				3		2						5
*Eptesicus serotinus*		4		35		20	15		5	4	15	98
*Nytalus leisleri*		1		5		1	1		1	1	1	11
*Nyctalus noctula*		1		39		21	3		18		6	88
*Pipistrellus kuhlii*	4				6					67		77
*Pipistrellus nathusii*		32		2	1		3		3			41
*Pipistrellus pipistrellus*	47	2	33	83						21	12	198
*Pipistrellus pipistrellus/pygmaeus*		8										8
*Pipistrellus pygmaeus*		32			3					45		80
*Pipistrellus savii*			2									2
*Pipistrellus* spp.	1	12										13
*Barbastella barbastellus*	6			4		3	2		10	3		28
*Plecotus auritus*	2			25			38	7	14		2	88
*Plecotus austriacus*	3		5	11			1				1	21
*Plecotus* spp.	4			4								8
*Vespertilio murinus*				3							1	4
*Myotis alcathoe*		1										1
*Myotis bechsteinii*	1			5		4						10
*Myotis brandtii*		1		1		1	6				1	10
*Myotis capaccinii*					1							1
*Myotis dasycneme*							2					2
*Myotis daubentonii*	14	19	18	17		9	62			3	5	147
*Myotis emarginatus*	7	2										9
*Mytois escalerai*										1		1
*Myotis myotis*	2			22		13	1				1	39
*Myotis mysticanus*	8			14	1	10	9					42
*Myotis nattereri*	2	1		7		6	12	37	6		2	73
*Myotis* spp.				3			8					11
*Miniopteris schreibersii*				1		1				1		3
*Not determined*	6	2		19			2		4		12	45

Individuals from high‐flying species represented 17% of all casualties (*n* = 206). These included species from the *Nyctalus*,* Eptesicus,* and *Vespertilio* genera. In two studies, authors were able to relate the number of casualties with local abundance estimates. They reported that *Nyctalus noctula* (Gaisler et al. 2009) and *Eptesicus serotinus* (Lesiński et al. 2010) formed a lower proportion of casualties than would be predicted from their relative abundance estimated by acoustic surveys (Gaisler et al. 2009) and netting (Lesiński 2007).

**Figure 1:**
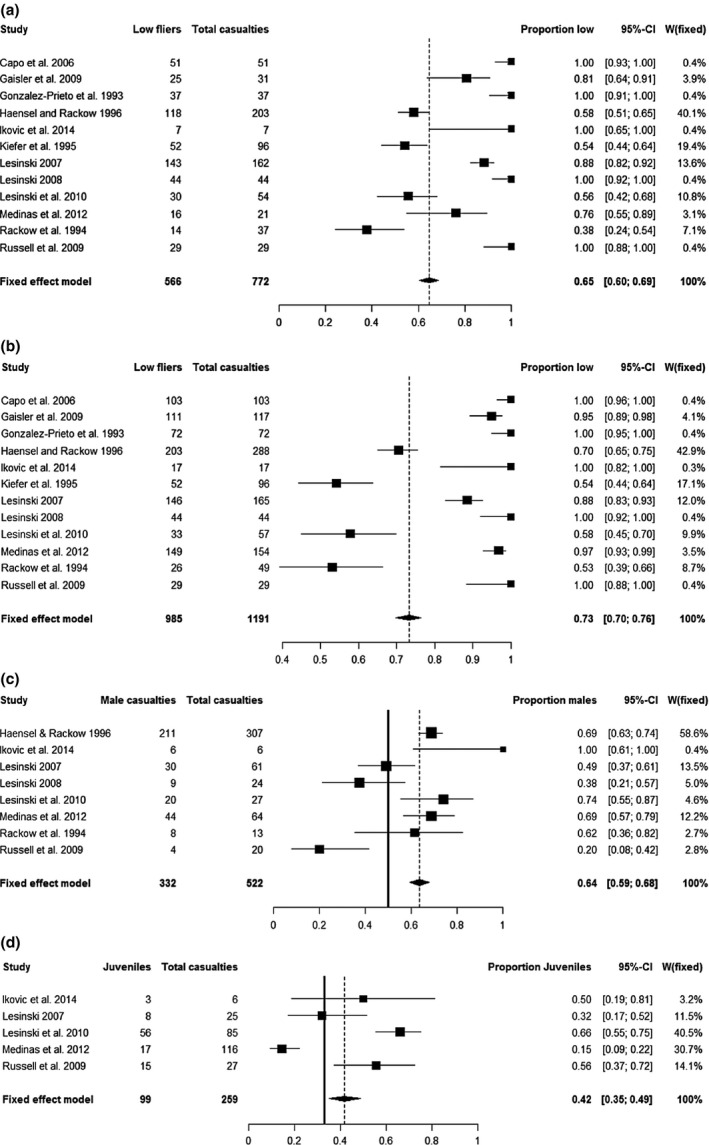
Forest plots showing the number of low‐flying bats, excluding (a) and including *Pipistrellus* individuals (b), male (c), and juvenile (d) casualties reported in each study, and the overall proportion of each category of casualties obtained from the combined data. A dotted line represents the overall proportion, a solid line represents the expected proportion. Figures 1a,b do not have a solid line as it was not possible to calculate an expected proportion. The width of the diamond denotes the confidence interval. ‘W(fixed)’ refers to the relative weight of each study under a fixed‐effect model.

Meta‐analysis demonstrated that most road casualties were attributed to low‐flying species (excluding *Pipistrellus* individuals *n* = 566, including *Pipistrellus* individuals *n* = 985; Fig. 1a,b, respectively). Sensitivity analysis showed that the exclusion of individual studies had little effect on this bias towards low‐flying species (Appendices S5 and S6). Of the 12 studies we identified during our systematic review, all but one documented a majority of low‐flying species, while a further five studies did not document any high‐flying species at all (Gonzalez‐Prieto et al. 1993, Capo et al. 2006, Lesiński 2008, Russell et al. 2009, Iković et al. 2014).

Meta‐analysis confirmed a highly significant overall bias towards males (332 males: 190 females; pooled confidence interval 0.59–0.68; Fig. 1c). The trend towards female casualties (16/20) observed in Russell et al.'s (2009) study is likely to be due to the proximity of the surveyed road to a known maternity roost. However, sensitivity analysis showed that the exclusion of individual studies had no effect on the overall bias towards males (see Appendix S7). Of the 12 studies we identified during our review, in eight, the sex of casualties was reported. In four, a significant bias towards males was reported (Haensel & Rackow 1996, Lesiński et al. 2010, Medinas et al. 2012, Iković et al. 2014); in one, there was a non‐significant trend towards males (Rackow et al. 1994). In one study, a significant bias towards females is reported (Russell et al. 2009) while in another, there is a non‐significant trend towards females (Lesiński 2008), and in one study, no significant deviation from an equal sex ratio is reported (Lesiński 2007).

Meta‐analysis showed that the observed proportion of juveniles was significantly higher than expected (pooled confidence interval 0.35–0.42), supporting our hypothesis that juvenile bats are more prone to collisions with vehicles than adults (Fig. 1d). Sensitivity analysis demonstrated that the bias towards juveniles was robust to the exclusion of individual studies; the mean proportion ranged from 0.34 to 0.54, indicating that the proportion of juveniles is always greater or equal to their expected abundance in the population (see Appendix S8). In five studies, information was included on the age of bat carcasses found on roads. Of these, in two, a significant bias towards juveniles was reported (Lesiński 2007, Russell et al. 2009), in another, a non‐significant bias towards juveniles was reported (Iković et al. 2014), while Lesiński et al. (2010) report no significant deviation from the expected proportion, and in one study a significant bias towards adults is reported (Medinas et al. 2012).

### Environmental risk factors for collision

In seven studies, the authors report that bat casualties were commonly found where roads were close to or bisected other linear features, including treelines (Lesiński 2008, Russell et al. 2009), hedges (Capo et al. 2006), rivers (Iković et al. 2014), viaducts (Medinas et al. 2012), forest edges, and woodland paths (Lesiński 2007, Lesiński et al. 2010, Table 1).

There is some evidence that the height and proximity of linear features to roads contribute to collision risk. For example, Russell et al. (2009) note that there were no carcasses where the canopy cover did not run adjacent to the road (within 15 m), but that mortalities were particularly high where the height of canopy declined close to the road. Lesiński et al. (2010) suggest that the relatively high occurrence of *Nyctalus noctula* mortalities found in their study may be due to the intersection of known forest flight paths, the proximity of the woodland to the road and the topology of the site. The combined effect of these ecological attributes may have encouraged *Nyctalus noctula* to cross the road at a lower height than is usual for this species (Lesiński et al. 2010).

The authors of four studies related bat mortalities to the quality of the habitat bordering roads. Medinas et al. (2012) report that bats were more likely to be killed where roads bisect high quality habitats or pass close to scarce and unevenly distributed foraging locations, such as water bodies and riparian habitats. This supports Gaisler et al.'s (2009) and Lesiński's (2007) findings that *Myotis daubentonii* carcasses were found in high numbers near water bodies. Likewise, Iković et al. (2014) found that casualties of *Pipistrellus* species were predominantly clustered at two points where the focal road crossed two tributary rivers.

We proposed that roads could pose a collision risk where they provide foraging opportunities for bats. The systematic review revealed that few researchers have explored this issue. However, the presence of woodland species such as *Barbastella barbastellus* and *Rhinolophus hipposideros* on roads could indicate that they are foraging along roads as they would along woodland paths. Lesiński (2007) suggests that exceptionally high numbers of *Myotis daubentonii* casualties could result from young inexperienced bats mistaking damp road surfaces for the surface of water.

Where a comparison could be made, bat road casualties were more common at locations with greater traffic volume. Iković et al. (2014) report that of the 17 bats retrieved from two roads bordered by similar habitat, 16 (0.8/km) were found on the road with relatively high traffic volume (10300 vehicles per day), while just one was found on the road with low traffic volume (1100 vehicles per day). Medinas et al. (2012) report a significant difference in the number of road‐killed bats on different types of road: the average number of carcasses found during their survey period was highest (3.99 ± 0.83/km) on the road with the greatest nightly traffic volume (1210 vehicles per night), a little lower (3.60 ± 0.89/km) on a road with intermediate traffic volume (277 vehicles per night), and very low (1.00 ± 0.30/km) on the quietest rural roads (<100 vehicles per night).

## Barrier Effects

### Risk factors associated with the behaviour and ecology of bats

Meta‐analysis reveals that flight height influenced the tendency of bats to avoid roads, as we predicted; some support for the influence of foraging strategy was provided by a qualitative review of the literature. Bennett and Zurcher (2013) report that the higher a bat flies as it approaches the road the more likely it is to cross (Appendix S9). *Myotis bechsteinii*, which forages largely by gleaning, was found to be far less likely than *Barbastella barbastellus* (which forages by hawking) to cross a motorway, and only crossed at underpasses (Kerth & Melber 2009). Low flying, clutter‐adapted *Myotis* spp. and *Plecotus auritus* passed over the road much less frequently than faster, open‐edge‐adapted species (e.g. *Pipistrellus* spp.) or high‐flying species (e.g. *Nyctalus leisleri;* Abbott et al. 2012b), and preferred to use less exposed river bridges and underpasses to cross rather than flying directly over the road (Abbott et al. 2012a). Where individuals of *Myotis* spp. and *Plecotus auritus* did cross roads, they preferred following severed treelines to flying over bridges (Abbott et al. 2012a). Although species from the genus *Pipistrellus* were more inclined than *Myotis* to use over‐road routes, they similarly preferred severed treelines to exposed crossing points (Abbott et al. 2012a).

### Environmental risk factors for barrier effects

The presence or absence of trees and shrub layers were influential factors in determining whether bats crossed roads; the presence of either tended to increase the likelihood of road‐crossing behaviour (Abbott et al. 2012a, Bennett & Zurcher 2013).

Bennett and Zurcher (2013) monitored road‐crossing behaviour at 10 known bat commuting routes bisected by a rural two‐lane road, and conducted classification and regression tree analyses to determine which predictor variables, alone or in combination, influenced crossing behaviour in the presence and absence of vehicles. In the presence of vehicles, the classification tree demonstrated a good performance with a reasonable fit, and in the absence of vehicles the model demonstrated excellent performance and a good fit. In both the presence and absence of vehicles, the presence of treelines was the most influential variable. In the absence of vehicles, 14% (8/57) of bats crossed the road where there was not a treeline, compared to 79% (81/102) where there was a treeline. In the presence of vehicles this effect was more pronounced: just 3.5% (2/58) of bats crossed the road in the absence of a tree line, in contrast to 59% (34/58) in the presence of a treeline (Appendix S10). In the absence of vehicles, the size of the gap between a linear feature and the road was the second most influential variable influencing whether bats crossed or turned back. Fewer bats crossed (2/11, 18%) where there was a gap of >4.5 m than where there were smaller gaps of 2.5–4.5 m (2/5, 40%; Bennett & Zurcher 2013).

Although bat activity levels were lower for over‐road routes, all species recorded in the vicinity of the roads crossed at severed treelines (Abbott et al. 2012a). At two under‐road‐crossing points (underpasses and river bridges), high numbers of bats passed above the road, which the authors attributed to the presence of extensive tree canopies above the motorway at these locations (Abbott et al. 2012a).

Direct evidence for the influence of road width on crossing behaviour is lacking. However, two studies, one from the UK (Berthinussen & Altringham 2012b) and one from the USA (Kitzes & Merenlender 2014), suggest that bat activity declines with proximity to major roads. Bat activity was 3.5 times higher at a distance of 1600 m from a 6–7‐lane road (approximately 32 m wide) than at the road (Berthinussen & Altringham 2012b), and activity was twice as high at 300 m from a 2‐lane road (15 m wide; Kitzes & Merenlender 2014).

The authors of two studies explored the impact of traffic on the probability of road crossing. Bennett and Zurcher (2013) found that in the absence of vehicles, 56% (89/159) of bats aborted crossing attempts, but in the presence of vehicles this rose to 68% (74/107); it rose further still to 100% (34/34) if vehicles produced noise levels above 88 dB. Bats may be particularly sensitive to noise pollution because they use echolocation to hunt insect prey and to orientate themselves in their environment; *Myotis myotis* has been shown to avoid foraging in proximity to loud noise (Schaub et al. 2008), and foraging efficiency has also been observed to decline with proximity to traffic noise (Siemers & Schaub 2011). Zurcher et al. (2010) found that 32% (64/167) of bats aborted crossings in the absence of vehicles compared with 60% (29/44) in the presence of vehicles. It is unclear whether this effect is due to the influence of noise, vehicle headlights, or both.

## Discussion

We have identified substantial evidence indicating a significant risk to bats from roads, particularly through collision risk.

Our data indicate that casualties are more likely to be low‐flying than high‐flying bat species. Determining the likely impacts on populations is difficult in the absence of good data on population density. In Great Britain, the best available estimate indicates a ratio of low‐flying to high‐flying bats of of 0.97 including pipistrelles and 0.89 excluding pipistrelles (Harris et al. 1995, Battersby 2005). If these proportions are representative of mainland Europe, the casualty ratios of 0.73 (including pipistrelles) and 0.64 (excluding pipistrelles) found in this review suggest that while low‐flying species are the most common casualties, high‐flying bats are more frequently hit than would be expected from their relative population sizes.

A number of rare species such as *Barbastella barbastellus* and geographically restricted species such as those from the genera *Rhinolophus* and *Plecotus* were also found on roads. The presence of casualties from rare species on roads is of particular concern, as relatively low levels of additional mortality could potentially have an impact on the long‐term sustainability of local populations.

Meta‐analyses confirm significant biases towards juvenile and male casualties on roads. The higher number of male fatalities could be due to female‐biased philopatry and male‐biased dispersal, which are typical of mammal breeding systems (Greenwood 1980). Greater dispersal distances could mean that males encounter roads more often, and inexperienced sub‐adult males may be at particular risk. Males may also be more susceptible to collisions if they are more likely to roost or forage in the vicinity of roads: in many species, there is sexual segregation during the breeding season, and some evidence suggests that female bats occupy better quality habitats (Angell et al. 2013) or less fragmented habitat (Lintott et al. 2014) during this period. If and where roads represent, or are associated with, poor quality habitat (i.e. due to edge effects), it is possible that females are restricted to areas with lower road density.

Although our meta‐analysis shows that overall males have a higher casualty risk, in some locations there was excess mortality in females. This may be due to the proximity of a particular road to a maternity roost. Medinas et al. (2012) report male bias overall, but note that during early summer twice as many female as male carcasses were found on the roads, corresponding with the time of year when females form maternity roosts. The formation of maternity roosts in early summer could explain the second highest peak in mortalities which occurred in May (Appendix S4).

We identified considerable support for the hypothesis that the risk of collision increases at junctions between roads and other linear habitat features. Treelines running perpendicular to roads are preferred crossing points, and the proximity of tree stands and treelines to roads appears to increase the propensity of bats to cross roads. These features are also associated with mortality hotspots.

Unfortunately, it is difficult to draw firm conclusions about the effects of fatalities on local populations as they are rarely quantified. However, even low adult mortality may reduce effective population size in bats (Schorcht et al. 2009). This is particularly worrying given the presence of geographically restricted and locally vulnerable species such as *Plecotus auritus*. It is likely that the numbers reported underestimate true collision rates, as none of the researchers adjusted the observed casualty rates for observer efficiency and the removal of carcasses by predators. Removal or destruction of carcasses may significantly bias results, as carcasses do not persist for long on roads (Santos et al. 2011). For comparison, casualty rates at wind turbine sites are often considerably higher than the numbers of observed carcasses (see Huso 2011, Bernardino et al. 2012, Bispo et al. 2013).

Additional research is required to understand fully the factors influencing road‐crossing behaviour in bats. Most species have been shown to cross roads, but clutter‐adapted species, i.e. those species that are adapted to flight in woodland edges and interior, are least likely to do so. Where passages under roads are available, these are preferred to over‐road routes. Major roads appear to be more inhibitive than secondary roads, perhaps as a result of associated high traffic volumes on major roads. There is some evidence that roads with greater traffic densities are associated with higher collision rates between bats and vehicles. This apparent contradiction could be resolved with a better understanding of how different species respond to traffic, and perhaps more consistent reporting of traffic volume data.

Whether and how a particular road poses a threat to bats is species‐dependent; a given road could simultaneously pose a threat to some species as a result of collisions, and form an impermeable barrier to movement for other species. Some species' characteristics may increase the probability of both collision and barrier effects. For example, clutter‐adapted bat species are predicted to avoid roads, as they are associated with closed environments such as woodland interior. However, these species may sometimes commute or forage in small open spaces. It is possible that such species cross roads where the roads are narrow or where they are bordered by trees. The slow and low flight of clutter‐adapted species puts them at greater risk of collision with vehicles. Therefore, habitat fragmentation and collision risks may act in combination for some species.

A key challenge for bat conservation is resolving how to increase the permeability of roads to bats without increasing the likelihood of vehicle collision. Gantries are often included in the mitigation design of new roads. These structures span the road and, where possible, link linear traditional commuting features used by bats on either side of the road. Recent research suggests, however, that the few bats observed crossing at gantries do not increase the height at which they cross the road (Berthinussen & Altringham 2012a). Green bridges and under‐road passages should be explored as alternative forms of mitigation. Further research needs to be conducted on the efficacy of gantries and underpasses in relation to wider roads. Specifically, there is a need for more surveys of bat activity before and after road construction. Even where pre‐ and post‐construction surveys are conducted, different protocols and recording equipment are often used, and surveys often take place over a short period, making adjustments for the effects of factors such as weather extremely challenging.

Due to a lack of data, we were unable to investigate the influence of road age on collision risk or crossing behaviour of bats in this study. However, the age of a road could influence how bats interact with it, through either sensitisation (resulting in more pronounced avoidance behaviour and reduced collision risk over time) or habituation (more frequent crossing attempts and increased collision risk over time). The threat posed by a road could also appear to decline over time where local abundance of bats becomes suppressed as a result of collisions. The influence of road age on collision risk and crossing behaviour should be examined by means of long‐term, systematic pre‐ and post‐construction studies, where new roads or road adjustments are planned.

The influence of artificial lighting is likely to be one of the most significant factors determining how bats orientate themselves within the landscape, and whether roads present a barrier to movement (Hale et al. 2015, Mathews et al. 2015). To understand the relative influence of road characteristics on species' responses, future research should compare the effects on bats of roads that are lit with those that are unlit.

Bats are much less likely to cross roads in the presence of particularly loud vehicles (Bennett & Zurcher 2013). Further research is needed to understand the extent to which noise, specifically volume and frequency, effects road‐crossing behaviour. It is also possible that disorientation as a result of traffic noise could make bats susceptible to collisions.

The need to examine the impact of roads is pressing; road density is already extremely high in the UK and mainland Europe, and further development is expected in the near future. To ensure that appropriate and effective mitigation is incorporated during the planned period of intensive road expansion, it is essential that the factors influencing road‐crossing behaviour in bats are fully understood. A better understanding of how bats interact with roads of varying widths, traffic densities and lighting schemes, as well as the role of the surrounding topography and habitat, could contribute to the design of safe crossing points.

## Supporting information


**Appendix S1.** The flight height category to which each reported species included in the meta‐analysis was assigned.
**Appendix S2.** The total number of casualties reported by each country.
**Appendix S3.** Site characteristics as reported by authors.
**Appendix S4**. Line chart showing the numbers of casualties reported by each study at different times of the year.
**Appendix S5.** Sensitivity analysis for flight height (excluding *Pipistrellus*).
**Appendix S6.** Sensitivity analysis for flight height (including *Pipistrellus*).
**Appendix S7.** Sensitivity analysis for sex bias.
**Appendix S8.** Sensitivity analysis for age.
**Appendix S9.** Results reported by Bennett and Zurcher (2013) of the number of bats crossing in the presence and absence of vehicles and the height at which they were observed crossing.
**Appendix S10.** Results reported by Bennett and Zurcher (2013) of the number of bats crossing in the presence and absence of vehicles and the presence or absence of a tree layer.
