# Supplementary material for: Roads and bats: a meta‐analysis and review of the evidence on vehicle collisions and barrier effects
Source: Mamm Rev. 2016 May 29;46(4):311–23. doi: 10.1111/mam.12072 (PMC5031215; doi:10.1111/mam.12072)
Supplement: Supplementary file 1 — Appendix S1. The flight height category to which each reported species included in the meta‐analysis was assigned. Appendix S2. The total number of casualties reported by each country. Appendix S3. Site characteristics as reported by authors. Appendix S4. Line chart showing the numbers of casualties reported by each study at different times of the year. Appendix S5. Sensitivity analysis for flight height (excluding Pipistrellus). Appendix S6. Sensitivity analysis for flight height (including Pipistrellus). Appendix S7. Sensitivity analysis for sex bias. Appendix S8. Sensitivity analysis for age. Appendix S9. Results reported by Bennett and Zurcher (2013) of the number of bats crossing in the presence and absence of vehicles and the height at which they were observed crossing. Appendix S10. Results reported by Bennett and Zurcher (2013) of the number of bats crossing in the presence and absence of vehicles and the presence or absence of a tree layer. [file MAM-46-311-s001.pdf]

Supplementary Information

Appendix S1. The flight height category to which each reported species included in the meta-analysis was assigned.

Taxon	Flight height
Rhinolophus blasii	Low
Rhinolophus ferrumequinum	Low
Rhinolophus hipposideros	Low
Eptesicus nilsonii	High
Eptesicus serotinus	High
Nyctalus leisleri	High
Nyctalus noctula	High
Pipistrellus kuhlii	Low
Pipistrellus nathusii	Low
Pipistrellus pipistrellus	Low
Pipistrellus pygmaeus	Low
Pipistrellus savii	Low
Pipistrellus spp.	Low
Barbastella barbastellus	Low
Plecotus auritus	Low
Plecotus austriacus	Low
Plecotus spp.	Low
Vespertilio murinus	High
Myotis alcathoe	Low
Myotis bechsteinii	Low
Myotis brandtii	Low
Myotis capaccinii	Low
Myotis dasycneme	Low
Myotis daubentonii	Low
Myotis emarginatus	Low
Myotis escalerai	Low
Myotis lucifugus	Low
Myotis myotis	Low
Myotis mysticanus	Low
Myotis nattereri	Low
Myotis sodalis	Low
Miniopteris schreibersii	Low
Myotis spp.	Low

Appendix S2. The total number of casualties reported by each country. The number in brackets refers to the total number of studies conducted in that country.

Country	Total casualties	Combined length of	Total survey time	Casualties/km	Casualties/km/month

		roads surveyed (km)	(months)		
Czech Republic (1)	119	8	6	14.8	2.4
France (1)	104	2	24	52	2.1
Germany (3)	464				
Montenegro (1)	17	30	14.5	0.5	0.03
Poland (3)	225	25.6		8.7	1.4
Portugal (1)	154	51	3	3.	1
Spain (1)	72	17	12	4.2	0.3
USA (1)	29	0.1	3	193.3	64.4

Appendix S3. Site characteristics as reported by authors. This data was not available for three studies (Rackow et al. 1994, Kiefer et al. 1995, Haensel & Rackow, 1996) and so they have not been included.

**Reference**	**Number of roads**	**Road length**	**Road width**	**Traffic volume**	**Lit/unlit**	**Surrounding habitat type**
Capo et al. 2006 (France)	1	Not reported	Not reported	Not reported	Partly lit	Sparse vegetation, hedgerow and isolated trees.
Gaisler et al. 2009 (Czech Republic)	2	3.5 km and 4.5 km	Four lane	Not reported	Not reported	One road had a lake on either side, the other crossed over a stream.
Gonzalez-Prieto et al. 1993 (Spain)	1	17 km	Two lanes (10 m wide including hard shoulders)	Not reported	Not reported	Trees, vineyards and shrubland.
Iković et al. 2014 (Montengro)	2	20 km high-traffic road and 10 km low-traffic road	Not reported	One high-traffic road with 10300 vehicles per day and one low-traffic road with 1100 vehicles per day.	One of the monitored sites had streetlights	Meadows, wetlands and Mediterranean karst scrub.
Lesiński 2007 *Method 1* (Poland)	1	8 km	Two lanes	Heavy traffic	Not reported	Not reported
Lesiński 2007 *Method 2* (Poland)	Several	NA	Not reported	Not reported	NA	Not reported
Lesiński 2008 (Poland)	1	1 km	Four lanes	“heavy and round the clock”	Not reported	Allotment gardens on one side and open area on the other, urban

						development and a section of forest.
Lesiński et al. 2010 (Poland)	1	16.6 km	One lane (7 m)	"relatively intensive"	Not reported	71%, windbreaks and bushes, 3%; built-up area, 4%; meadows and pastures, 15%; and arable fields or wastelands, 7%"
Medinas et al. 2012 (Portugal)	Several	51 km transect	2 lanes on average	Ranged from 1500 per night on busiest road to <100 on quietest roads	Not reported	Primarily Mediterranean agro-forestry.
Russell et al. 2009 (Indiana, USA)	1	4.5 km	20 m wide	8569 vehicles per day (12% trucks)	Not reported	Few buildings and a large area of foraging habitat on opposite side of the road from the roost.

Appendix S4. Line chart showing the numbers of casualties reported by each study at different times of the year. Ten studies reported the season in which carcasses were retrieved.

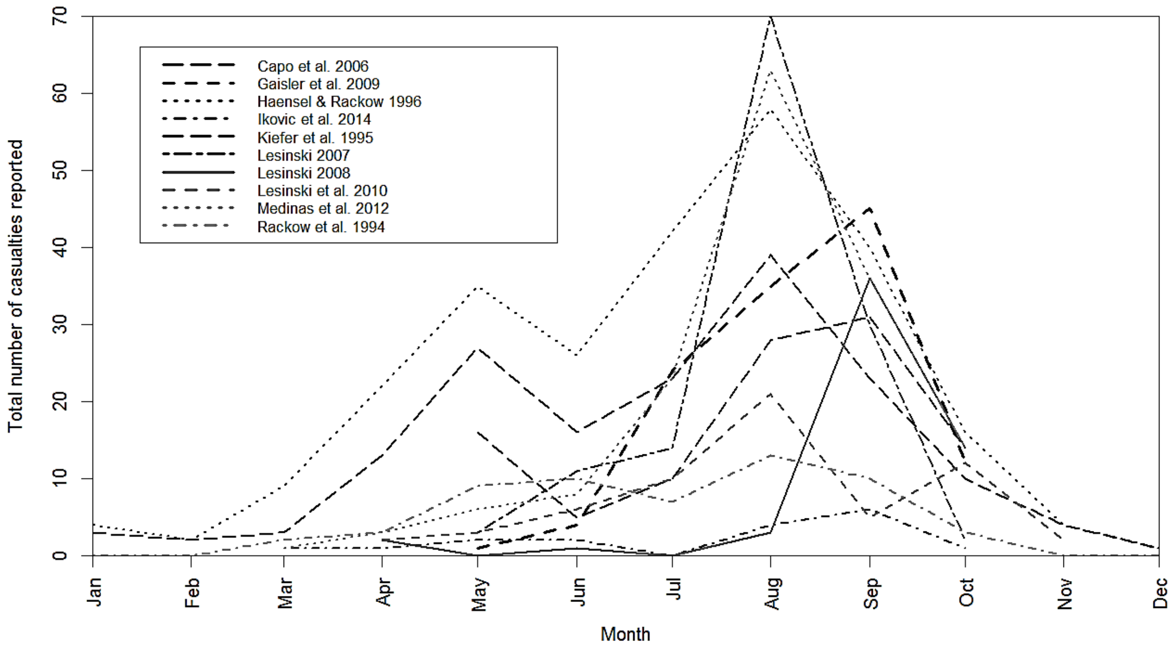

Appendix S5. Sensitivity analysis for flight height (excluding Pipistrellus).

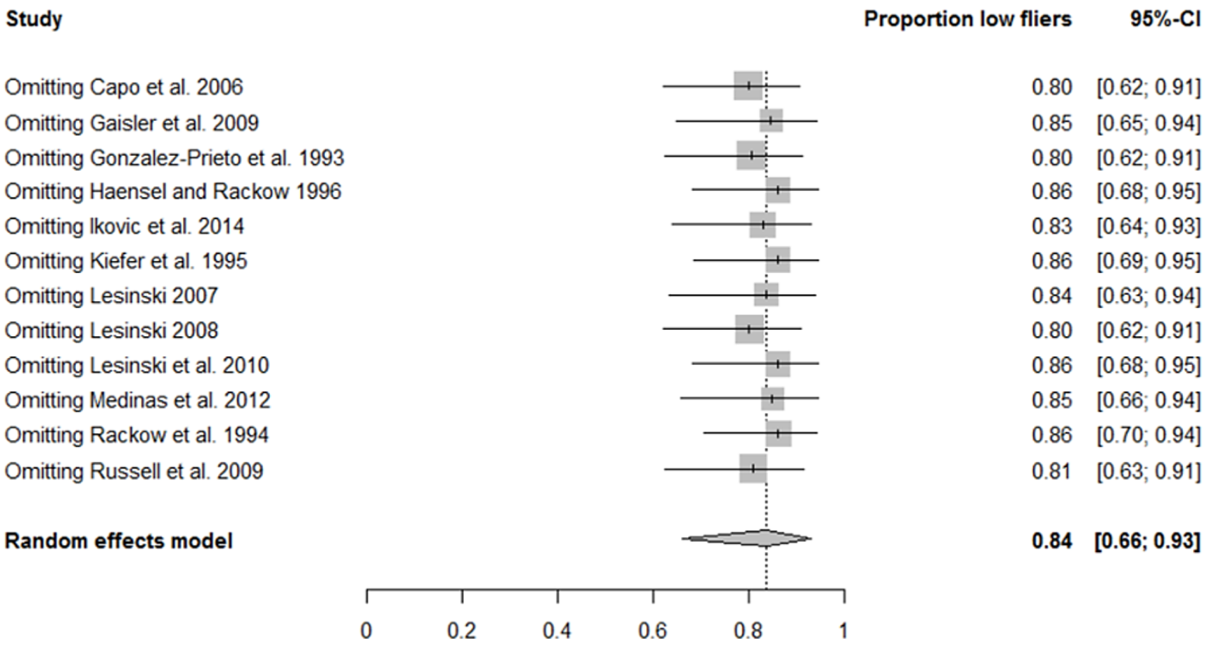

Appendix S6. Sensitivity analysis for flight height (including Pipistrellus).

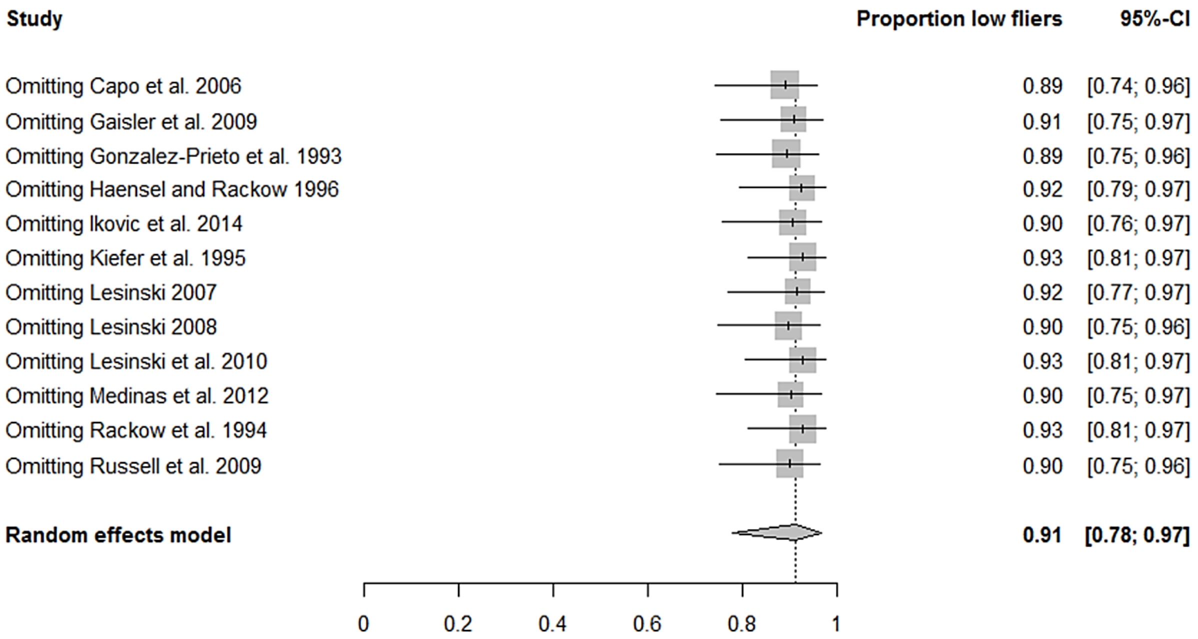

Appendix S7. Sensitivity analysis for sex bias.

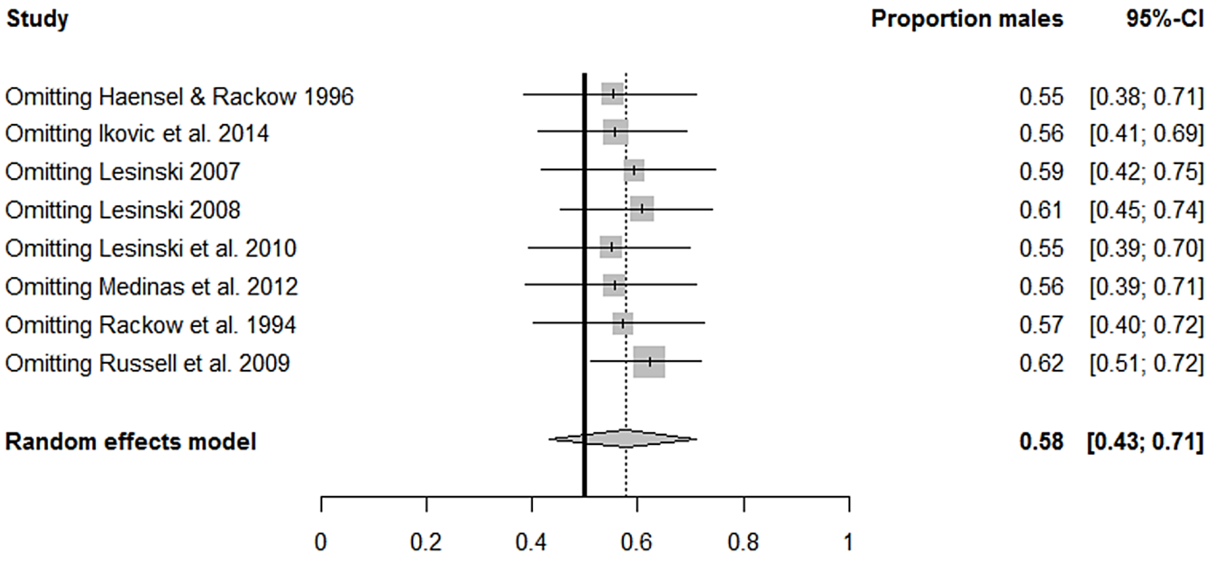

Appendix S8. Sensitivity analysis for age.

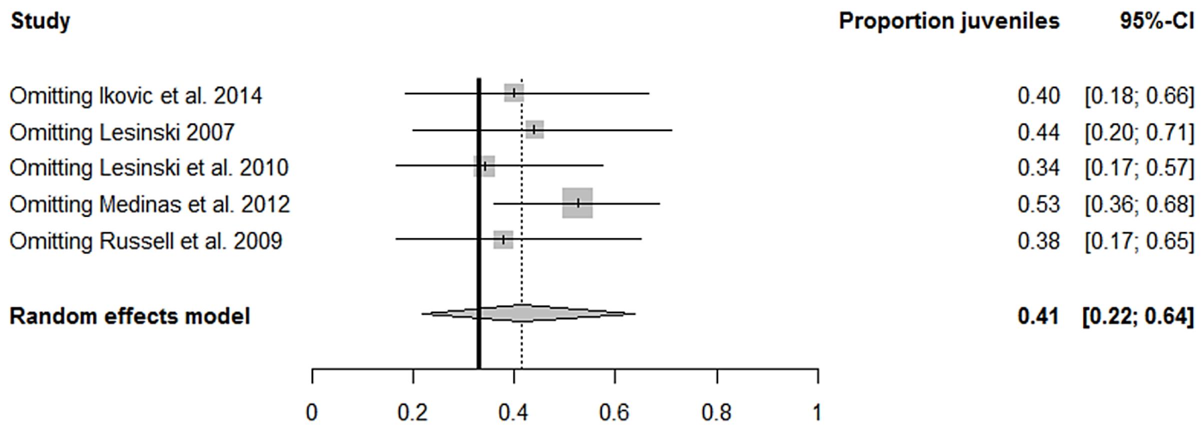

Appendix S9. Results reported by Bennett & Zurcher (2013) of the number of bats crossing in the presence and absence of vehicles and the height at which they were observed crossing.

Vehicles	Height (m)	Crossing
Absent	<7.5	37% (3/8)
Absent	>7.5<14	91% (43/47)
Absent	>14	100% (31/31)
Present	<9	57% (8/14)
Present	>9<13	72% (8/11)
Present	>13	90% (9/10)

Appendix S10. Results reported by Bennett & Zurcher (2013) of the number of bats crossing in the presence and absence of vehicles and the presence or absence of a tree layer.

Vehicles	Tree layer	Crossing
Present	Absent	3.45% (2/58)
Present	Present	58.62% (34/58)
Absent	Absent	14% (8/57)
Absent	Present	79% (81/102)
