# Supplementary material for: Roads and bats: a meta‐analysis and review of the evidence on vehicle collisions and barrier effects
Source: Mamm Rev. 2016 May 29;46(4):311–23. doi: 10.1111/mam.12072 (PMC5031215; doi:10.1111/mam.12072)
Supplement: Supplementary file 2 [file MAM-46-311-s002.docx]

**Supporting information**

**Appendix S1.** The flight height category to which each reported species included in the meta-analysis was assigned.

**Appendix S2.** The total number of casualties reported by each country. The number in brackets refers to the total number of studies conducted in that country.

**Appendix S3.** Site characteristics as reported by authors. This data was not available for three studies (Rackow et al. 1994, Kiefer et al. 1995, Haensel & Rackow, 1996) and so they have not been included.

**Appendix S4**. Line chart showing the numbers of casualties reported by each study at different times of the year. Ten studies reported the season in which carcasses were retrieved.

**Appendix S5.** Sensitivity analysis for flight height (excluding Pipistrellus).

**Appendix S6.** Sensitivity analysis for flight height (including Pipistrellus).

**Appendix S7.** Sensitivity analysis for sex bias.

**Appendix S8.** Sensitivity analysis for age.

**Appendix S9.** Results reported by Bennett & Zurcher (2013) of the number of bats crossing in the presence and absence of vehicles and the height at which they were observed crossing.

**Appendix S10.** Results reported by Bennett & Zurcher (2013) of the number of bats crossing in the presence and absence of vehicles and the presence or absence of a tree layer.
